# Effects of soil layer replacement combined with organic fertilizer application on soil quality and maize yield in heavy metal-contaminated farmland

**DOI:** 10.1016/j.isci.2026.116381

**Published:** 2026-06-12

**Authors:** Huanhuan Wang, Han Tu, Ping Wang, Xuexian Li, Pan Wu

**Affiliations:** 1 College of Agriculture, Guizhou University, Guiyang 550025, China; 2 Key Laboratory of Karst Georesources and Environment of Ministry of Education, Guizhou University, Guiyang 550025, China; 3 Guizhou Xingshuo Mingyue Environmental Protection Technology Co., Ltd, Guiyang 550025, China; 4 Rural Energy and Environment Agency of Guizhou Province, Guiyang 550025, China

**Keywords:** Soil science, Environmental science, Pollution, Soil chemistry

## Abstract

High geological background, coupled with mining activities, cause heavy metal enrichment in karst topsoil. A field experiment was carried out with two soil replacement depths (T0: 0–20 and 20–40 cm; T1: 0–30 and 30–60 cm), combined with three application rates of biochar, cow manure, and liquor brewing sludge-derived organic fertilizer. Soil physical property index (SPI), soil chemical property index (SCI), soil microbial property index (SMI) and soil quality index (SQI) were adopted to evaluate soil improvement, heavy metal reduction, and maize yield. The results showed that T1 reduced Cd, Pb, and Zn by 45.8%–71.1%, complying with GB 15618 2018 limits. The high-concentration liquor brewing sludge-derived organic fertilizers (CYH) achieved the highest SQI value. Partial least-squares path modeling verified SQI as the primary yield determinant. Overall, T1 + CYH is an effective strategy for heavy metal mitigation, soil quality improvement, and maize yield promotion. These findings provide scientific support for the safe utilization of heavy metal-contaminated karst farmland.

## Highlights

• Soil replacement at 0–30 and 30–60 cm effectively reduces heavy metal pollution in topsoil
• Liquor brewing sludge-derived organic fertilizers can greatly improve overall soil quality
• Optimized soil chemical properties mainly promote soil quality and boost crop yields
• Combined T1 + CYH treatment realizes pollution control, soil improvement, and yield promotion

## Graphical abstract

**Figure:**
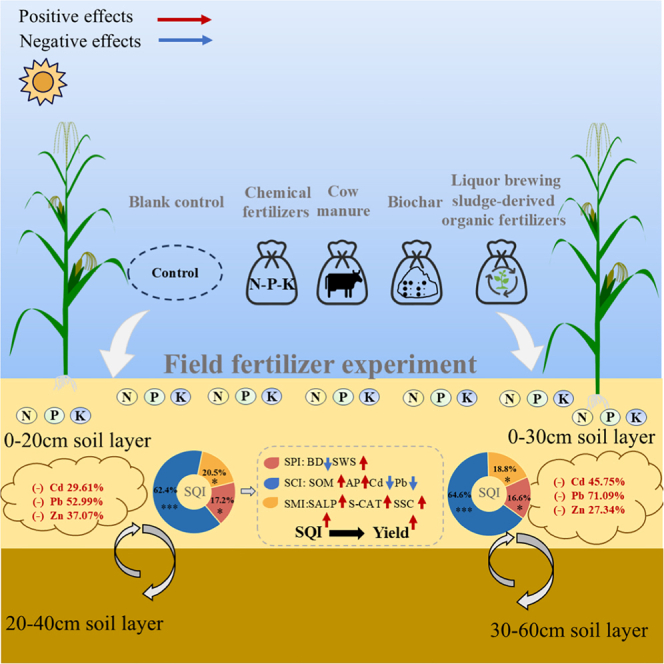

## Introduction

Soil serves as a fundamental basis for human survival and material production.^1^^,^^2^^,^^3^ Soil quality is pivotal for global food security, ecological stability, climate change mitigation, and biodiversity conservation.^1^^,^^2^^,^^4^ However, the combined impact of intensifying human activities and the natural high geological background in karst regions has resulted in pronounced heavy metal accumulation in soil.^5^^,^^6^ Currently, soil heavy metal pollution has emerged as a pressing global environmental challenge. Reports indicate that 14%–17% of farmland soil worldwide is affected by heavy metal contamination,^6^ posing substantial threats to human health, food security, and ecosystem integrity. Heavy metal pollution in soil can induce soil compaction and nutrient depletion, inhibit soil enzyme activity, and disrupt the structure and function of microbial communities, thereby compromising soil quality.^7^^,^^8^ Concurrently, it reduces crop yields, jeopardizes food safety, and even endangers human health through the food chain, thus garnering extensive global concern.^7^^,^^9^

Owing to the unique geological backdrop of karst regions, the weathering of carbonate rocks into soil triggers the secondary enrichment of heavy metals, resulting in elevated background values of soil heavy metals.^5^ Meanwhile, these regions have abundance of energy and mineral resources. With the intensification of regional mining and metallurgical activities, heavy metals have become significantly concentrated in the soil surface layer.^10^ Additionally, soils in karst areas are characterized by shallow and discontinuous layers, low fertility, and heavy texture.^5^^,^^11^ This “congenital deficiency” not only exacerbates the scarcity of cultivated land resources in karst regions but also compounds the detrimental effects of heavy metal pollution from mining and metallurgy, severely deteriorating regional soil environmental quality.^11^^,^^12^ The safety of cultivated land and the quality of agricultural products are put at risk, posing a major obstacle to arable land productivity and constraining the sustainable development of regional agriculture.

Currently, in agricultural production, chemical *in situ* passivation and biological and microbial remediation technologies are primarily employed to tackle severe heavy metal pollution in cultivated land soil, aiming to ensure the safe utilization of arable land.^13^^,^^14^ However, these methods immobilize heavy metals only in the plow layer soil, rather than reducing their total concentrations. Concerns persist regarding the long-term stability and safety of soil passivators and amendments. Moreover, heavy metals stabilized under certain conditions may be re-released when soil environmental factors change,^15^ potentially triggering “secondary” pollution. Biological and microbial remediation approaches are hampered by their lengthy treatment cycles, slow efficacy, and stringent ecological requirements,^14^^,^^16^ which limit their practical application. In response to the surface-concentrated nature of soil heavy metal pollution, scholars have proposed soil layer replacement technology.^14^^,^^17^ This technique mechanically replaces contaminated topsoil with cleaner deep soil layers to dilute pollutants.^17^^,^^18^^,^^19^ For example, Wang et al.^18^ reported that exchanging 0–30 cm topsoil with 30–60 cm subsoil reduced plow layer cadmium (Cd) from 3.19 mg/kg to 1.96 mg/kg. Yang et al.^19^ also found that soil layer replacement decreased mercury (Hg), zinc (Zn), and copper (Cu) concentrations by 28.09%, 2.31%, and 6.18%, respectively. In addition, soil layer replacement alleviates topsoil acidification,^20^ representing an effective strategy to remediate surface soil pollution.

Although soil layer replacement can effectively reduce heavy metal concentrations in the plow layer, replacing nutrient-rich topsoil with subsoil often results in a considerable decrease in soil fertility.^19^^,^^21^ For this reason, organic fertilizer amendment has been widely adopted to restore a fertile and functional plow layer.^22^ Organic fertilizers generally improve soil physical, chemical, and biological properties by enhancing soil aeration, retaining nutrients, and replenishing organic matter, which, in turn, provide a favorable environment for crop growth.^23^^,^^24^^,^^25^ For example, livestock poultry manure can significantly increase the soil organic carbon concentration and promote its accumulation in soil macro-aggregates, thus contributing to higher crop yields.^3^^,^^24^ Biochar can adsorb nutrient ions through its porous structure and large specific surface area, thereby reducing nutrient leaching and volatilization.^25^ Accordingly, both livestock poultry manure and biochar have been widely applied to improve soil fertility and crop productivity.

The liquor industry serves as a vital pillar of China’s economy, making significant contributions to the country’s economic development. However, large amounts of brewing sludge are generated annually, and their safe disposal remains a key challenge. Liquor sludge is mainly derived from crop-based raw materials, with low levels of heavy metals and hazardous substances, and is rich in organic matter, starch, protein, lignocellulose, and mineral nutrients, making it a promising resource for agricultural applications.^25^^,^^26^^,^^27^ Composting can effectively convert liquor sludge into qualified organic fertilizers that meet the Standards for Pollution Control of Sludge for Agricultural Use (GB 4284-2018) and the industry standard Organic Fertilizer (NY/T 525–2021). Such recycling reduces environmental pressure and improves the value of solid waste, consistent with the concept of the circular economy. Modified liquor-sludge organic fertilizer can be widely used for soil amelioration, fertility improvement, and ecological restoration of contaminated or low-yield farmland.^25^^,^^27^

To evaluate the effects of soil layer replacement combined with organic fertilizer application on heavy metal reduction and topsoil fertility restoration, a comprehensive soil quality assessment is essential. Soil is a complex ecosystem governed by interactive biological, mineral, and microbial processes, and its properties are highly sensitive to environmental changes.^1^^,^^28^ However, single soil indicators cannot fully reflect overall soil quality, highlighting the demand for integrated and reliable evaluation methods.^2^^,^^29^ The soil quality index (SQI) is a systematic framework based on soil physical, chemical, and microbiological properties, which is invaluable for evaluating multiple soil functions, including fertility, environmental quality, and ecological health.^4^^,^^30^^,^^31^ Its evaluation system primarily encompasses key parameters such as soil organic matter (SOM) concentration, nutrient status, texture and bulk density (BD), pH, and soil enzyme activity.^29^^,^^31^^,^^32^ However, when the traditional SQI integrates all indicators for evaluation, it struggles to accurately analyze interrelationships between indicators, potentially leading to biases in identifying the drivers of soil quality changes.^2^^,^^33^^,^^34^ Therefore, we propose to construct soil physical property index (SPI), soil chemical property index (SCI), and soil microbial property index (SMI) based on the calculation logic of the traditional SQI.^2^ By quantifying the indicators reflecting soil physical, chemical, and microbial properties, we aim to identify key factors influencing soil quality and thereby enable a more comprehensive and scientific assessment.

Additionally, in karst regions with superimposed mining and metallurgical activities, heavy metal pollution has emerged as a critical factor affecting soil quality.^5^^,^^10^ Excessive heavy metals can disrupt soil physicochemical properties and microbial communities, thereby impairing soil structure and function.^4^^,^^7^^,^^9^ Therefore, soil heavy metal concentrations are equally vital for comprehensive and accurate soil quality assessment. Because heavy metals are, essentially, chemical elements in soil, their concentrations can directly reflect the concentration level of such chemical substances in soil. The soil heavy metal concentration index can be incorporated into the indicator system of the SCI. Although crop yield has not been fully integrated into conventional soil quality evaluation systems, it serves as a comprehensive reflection of soil fertility and production functions, thus remaining a critical consideration.^29^

Northwestern Guizhou is an important karst distribution area in Southwest China and exhibits unique geochemical high-background heavy metal characteristics.^6^^,^^35^^,^^36^ This region is rich in mineral resources and has a long history of mining activities. The combination of a high geological background and intensive smelting activities has caused serious heavy metal accumulation in topsoil,^37^ which greatly limits the improvement of soil quality and crop production. This study conducted field experiments in a typical heavy metal-polluted farmland in northwest Guizhou. A comprehensive evaluation system was constructed based on soil properties, including SQI, SPI, SCI, and SMI, to assess the effects of the combined application of soil layer replacement technology and organic fertilizer on heavy metals, soil quality, and maize yield. The specific objectives are as follows: (1) to evaluate the effect of soil layer replacement on reducing heavy metals in plow layer soil; (2) to assess the contributions of soil physical, chemical, and microbial characteristics to SQI; (3) to identify key driving factors for SQI and maize yield under the combined application of soil layer replacement and organic fertilizers. The research findings hold significant guiding value for exploring safe utilization strategies of cultivated land contaminated by heavy metals in karst regions and constructing healthy soil ecosystems.

## Results and discussion

### Soil physical properties

**Figure 1:**
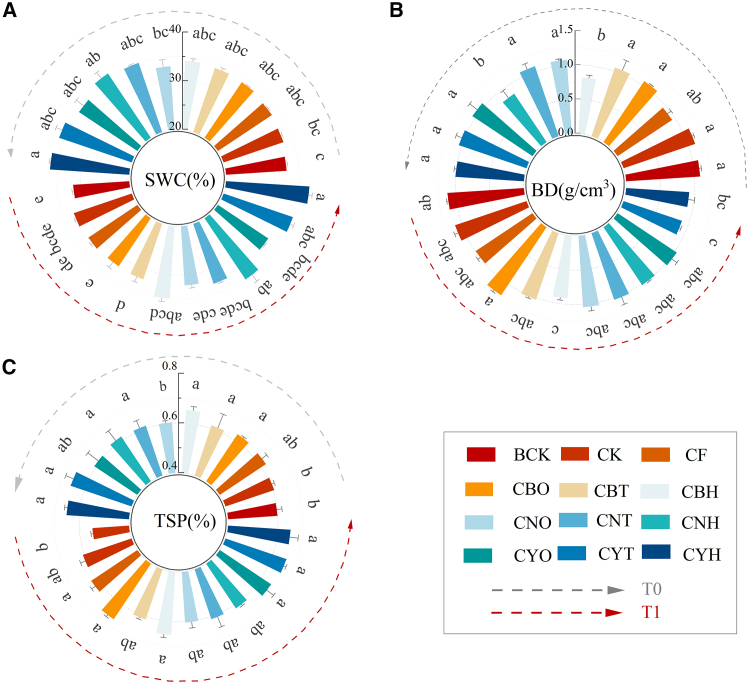
Effects of soil layer replacement combined with organic fertilizer application on soil physical properties (A–C) Soil water content (A), bulk density (B), and total porosity (C) under different treatments. Data are presented as mean ± standard deviation (SD). Different lowercase letters indicate significant differences among treatments at *p* < 0.05. All treatment abbreviations are defined as follows: BCK (non-replacement treatment), CK (replacement without fertilization), CF (chemical fertilizers), CBO (low-concentration biochar), CBT (medium-concentration biochar), CBH (high-concentration biochar), CNO (low-concentration cow manure), CNT (medium-concentration cow manure), CNH (high-concentration cow manure), CYO (low-concentration liquor-brewing sludge organic fertilizers), CYT (medium-concentration liquor-brewing sludge organic fertilizers), and CYH (high-concentration liquor-brewing sludge organic fertilizers).

Soil layer replacement combined with the application of organic fertilizers exerts a significant impact on soil physical properties (Figure 1). Under the condition of soil layer replacement at the 0–20 and 20–40 cm soil depths (T0), relative to those under the blank control (CK) and chemical fertilizer (CF) treatments, the soil physical properties under the treatment with organic fertilizer application were significantly improved. This phenomenon can be attributed to the strong adsorption capacity of the organic colloids in bio-organic fertilizers. These colloids can adsorb a large quantity of water, enhance the soil’s water-holding capacity, and effectively improve the soil water content (SWC), BD, and total porosity (TSP).^23^ Moreover, the organic matter in organic fertilizers can easily undergo transformation under the catalytic action of microorganisms, forming humus and other colloidal substances. These substances play a vital role in increasing the soil porosity, refining the arrangement of soil particles, and consequently reducing the soil BD.^38^ However, the improvement effect on soil physical properties exhibits significant differences depending on the source of organic fertilizers. In contrast to CK treatment, the CBH treatment significantly reduced the BD (25.62%) and significantly increased the TSP (7.00%) (Figures 1B and 1C). It exhibited favorable soil physical properties. This can be attributed to the fact that biochar is produced through the pyrolysis and carbonization of biomass under anaerobic or hypoxic conditions, and it has a rich pore structure.^25^^,^^39^ When biochar is applied to the soil, these pores become part of the soil pore system, directly increasing the TSP of the soil. At the same time, the pores in biochar prevent soil particles from packing closely together, thus reducing the BD.^39^^,^^40^

Under the condition of soil layer replacement at the 0–30 and 30–60 cm soil depths (T1), each treatment’s promoting effect on SWC is as follows: CYH > CNH > CYT > CBH > CNT > CK > CYO > CYO > CNO > CF > CBT > CBO > BCK. Compared with the non-replacement control treatment (BCK), the liquor brewing sludge-derived organic fertilizer (CYH) treatment significantly increased the SWC by 17.69% (Figure 1A, *p* < 0.05). Liquor brewing sludge-derived organic fertilizers are rich in various beneficial microorganisms. After entering the soil environment, these microorganisms continuously produce a large amount of secondary metabolites (such as humic acid compounds, extracellular polysaccharides, etc.) during their growth and reproduction process. These secondary metabolites can optimize the composition and distribution characteristics of soil pores, while simultaneously enhancing the water retention capacity of the soil. Compared with the BCK, the soil physical properties of each treatment were significantly improved under both substitution methods. This is because soil replacement led to a significant improvement in the soil compaction condition, a careful optimization of the pore distribution pattern, and a substantial enhancement of soil’s aeration capacity and ability to retain fertilizers.^20^

The above results demonstrate that soil layer replacement combined with organic fertilizer application effectively improves soil physical properties and water retention capacity. However, the practical application of this technology in karst regions still faces certain constraints. Limited by the shallow soil depth, discontinuous spatial distribution, and complex underlying bedrock structure in karst areas, the feasibility of large-scale mechanized soil layer replacement, to a certain extent, remains low. Excessively intensive replacement operations may disrupt the fragile epikarst zone, thereby affecting subsurface hydrological processes and increasing the risk of soil erosion.^41^ Therefore, the popularization and application of this technology should be carefully evaluated based on local topography and geological conditions. It is recommended that precise operations be preferentially implemented in relatively flat and contiguous farmland areas, and reasonable fertilization should be carried out according to the physical and chemical properties of the soil after replacement, so as to strike a balance between remediation benefits and ecological protection.

### Soil heavy metals and soil chemical properties

#### Soil heavy metals

**Figure 2:**
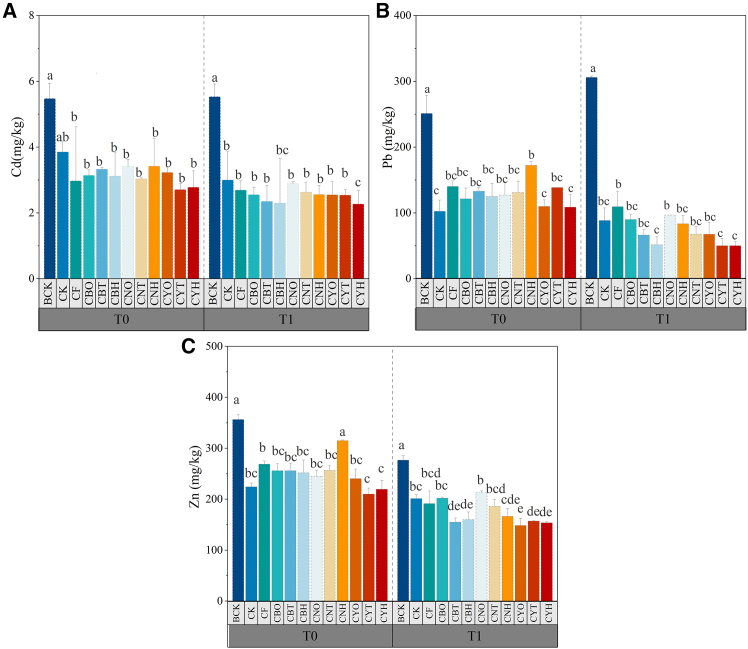
Effects of soil layer replacement combined with organic fertilizer application on heavy metal concentrations (A–C) Effects of soil layer replacement combined with organic fertilizer application on the concentrations of cadmium (A), lead (B), and zinc (C). Data are presented as mean ± standard deviation (SD). Different lowercase letters indicate significant differences among treatments at *p* < 0.05.

The cumulative effect of soil layer replacement combined with the application of organic fertilizers on soil heavy metals is shown in Figure 2. The results show that, under the two soil layer replacement modes, the heavy metal concentrations in the soil generally decreased. Under the T0 condition, compared with BCK, the concentrations of lead (Pb) and Zn under the remaining fertilization treatments were all within the range of the risk control values specified in the Risk Control Standards for Soil Pollution on Agricultural Land (GB 15618-2018). Notably, although cow manure is the most widely used organic fertilizer in the local area, the results show that under the condition of the same fertilization rate, the soil heavy metal concentrations in the cow manure treatments (CNO, CNT, and CNH) are generally higher than those in the liquor brewing sludge-derived organic fertilizer treatments (CYO, CYT, and CYH) and the biochar treatments (CBO, CBT, and CBH). This may be due to the fact that during the breeding process, cows consume a large quantity of feed additives containing heavy metal components. Cows ingest antioxidants with Cd and Zn, microbial agents, and other growth promoters, which are then excreted along with the cow manure (Figure 2).^42^ Therefore, on the premise of ensuring soil fertility supply, it is recommended to encourage local farmers to reasonably adopt pretreatment technologies, such as high-temperature composting combined with passivators and graded screening, to achieve the goal of effectively reducing heavy metal concentrations in cow manure. The southwest karst region is severely polluted by Cd due to the combined effect of a high geological background and anthropogenic sources.^12^^,^^30^ Compared with that in the BCK group, the Cd concentration in the soil treated with only soil layer replacement without fertilization (CK) decreased by 29.62%. After the application of organic fertilizers, the Cd concentration in different treatments ranged from 2.71 to 3.42 mg/kg. Only in the two treatments CYT and CYH were the Cd concentrations within the strictly controlled range (2.71 and 2.78 mg/kg, respectively) (Figure 2A), which was closely related to the relatively low heavy metal concentrations inherent in the liquor brewing sludge-derived organic fertilizers. The Pb concentration in different fertilization treatments ranged from 108 to 172 mg/kg, and the Zn concentration ranged from 210 to 256 mg/kg (Figures 2B and 2C).

Under the T1 condition, the concentrations of Cd, Pb, and Zn in the soil under each treatment were all lower than the risk control values specified in the standards for risk intervention value (GB 15618-2018) (Figure 2). The concentrations of heavy metals in the treatment groups applied with liquor brewing sludge-derived organic fertilizers were generally lower than those in the biochar treatment groups and the cow manure treatment groups. This could be attributed to the fact that the liquor brewing sludge-derived organic fertilizer adopts a continuous tank fermentation process, which can ensure sufficient decomposition and stable properties of raw materials, thereby potentially further reducing the bioavailability of residual heavy metals. Meanwhile, its abundant organic matter can enhance the complexation between Cd and humus, promoting the conversion of Cd into stable organic-bound fractions and reducing its mobility and bioavailability. In addition, the organic fertilizer itself has a relatively high pH value (Table S1), and this alkaline condition may facilitate the precipitation of Cd in the form of hydroxide or carbonate, further decreasing its bioavailability.^27^^,^^43^ Compared with BCK, the concentrations of Cd, Pb, and Zn in the CK decreased by 45.75%, 71.09%, and 27.35%, respectively; moreover, this reduction magnitude was higher than that of the corresponding treatment under T0 conditions. Compared with T0, T1 could more effectively reduce the main Cd, Pb, and Zn pollutants in the regional tillage soil. On the one hand, deeper soil layers generally contain lower heavy metal concentrations, and their replacement to the surface can effectively dilute heavy metal concentrations in the plow layer. On the other hand, deep tillage can more thoroughly break up the plow pan and improve the subsoil structure and porosity. Such optimization of the physical environment promotes plant root growth and alters the rhizosphere microenvironment, thereby indirectly enhancing the immobilization of heavy metals.

**Figure 3:**
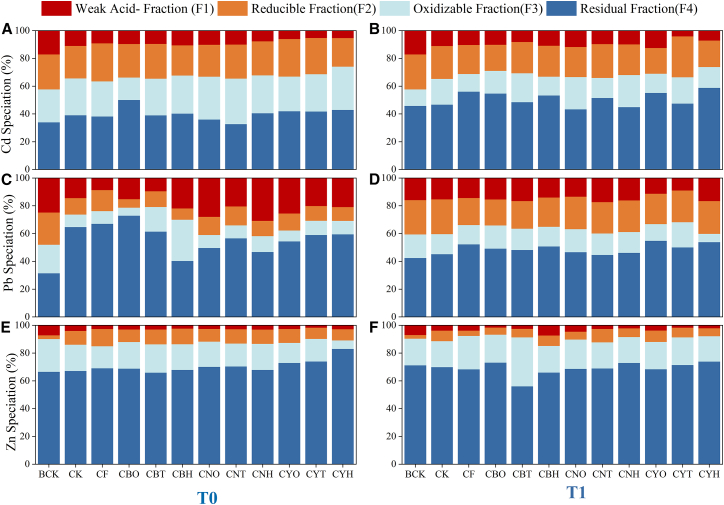
Effects of soil layer replacement combined with organic fertilizer application on soil heavy metal speciation (A) Effects of T0 tillage on Cd speciation. (B) Effects of T1 replacement on Cd speciation. (C) Effects of T0 replacement on Pb speciation. (D) Effects of T1 replacement on Pb speciation. (E) Effects of T0 replacement on Zn speciation. (F) Effects of T1 replacement on Zn speciation. Data are presented as mean ± standard deviation (SD). Different lowercase letters indicate significant differences among treatments at *p* < 0.05.

Heavy metal speciation is a critical indicator for evaluating heavy metal bioavailability. Heavy metals in soil mainly exist in four fractions— weak acid-extractable (F1), reducible (F2), oxidizable (F3), and residual (F4). Among them, F1 and F2 exhibit high mobility and bioavailability, being readily absorbed by plants.^44^ F3 is relatively stable, with potential release under strong oxidizing conditions, showing higher stability than F1 and F2. F4 is the most stable fraction, with extremely low mobility and transformation capacity, making it hardly bioavailable for plant uptake.^45^ The effects of tillage combined with organic fertilizer application on soil heavy metal speciation are presented in Figure 3. All three heavy metals (Cd, Pb, and Zn) were found to be predominantly present in the F4, with the stability ranking as follows: Zn (85.08%–89.86%) > Pb (50.04%–68.29%) > Cd (57.52%–71.20%). Cd exhibited the highest proportion of labile fractions (F1 + F2), indicating the highest bioavailability risk. Comparison between the two tillage modes revealed that the T1 tillage treatment resulted in a generally higher proportion of F4 and a lower proportion of labile fractions across all treatments compared with the T0 treatment, suggesting that deep tillage effectively promotes the transformation of heavy metals to stable fractions. Different organic fertilizers exert distinct effects on heavy metal speciation; treatments with liquor brewing sludge-derived organic fertilizer (CYO, CYT, and CYH) effectively increased the F4, leading to a marked reduction in the proportion of labile fractions of Cd, Pb, and Zn relative to the BCK treatment, demonstrating a prominent stabilization effect. In contrast, cow manure treatments tended to increase the proportion of labile fractions, posing a potential risk of heavy metal activation. In conclusion, T1 tillage combined with liquor brewing sludge-derived organic fertilizer application represents an ideal strategy to effectively reduce heavy metal bioavailability and enhance soil heavy metal stability.

Although this study confirms that the integrated remediation strategy can effectively reduce the total concentration and bioavailability of heavy metals in soil, the potential risk of heavy metal reactivation under fluctuating environmental conditions warrants further investigation. Processes such as organic matter decomposition, acid deposition, or extreme climate events may alter the chemical speciation and bioavailability of residual heavy metals.^45^^,^^46^ Specifically, during the decomposition of organic matter, small-molecule organic acids produced in the early and middle stages can form soluble complexes with heavy metal ions in the soil, and the decomposition products can regulate soil pH at the same time; these two factors act synergistically to promote the desorption and activation of fixed heavy metals in the soil. In the late stage of decomposition, if the humus structure is disrupted by external factors, the heavy metals it retains may also be released, thereby increasing the risk of heavy metal reactivation and secondary release. In addition, a decrease in soil pH tends to promote the transformation of stable heavy metal fractions (such as residual fractions) into forms with higher mobility and bioavailability; meanwhile, climatic factors (such as mean annual temperature [MAT] and mean annual precipitation [MAP]) can further regulate the bioavailability of heavy metals by controlling soil moisture status and leaching intensity.^46^^,^^47^ Therefore, to elucidate the long-term sustainability of this remediation technology, future studies should conduct long-term *in situ* monitoring experiments to systematically evaluate the evolution of heavy metal chemical speciation under different environmental scenarios and verify the long-term stability of the passivation effect.

#### Soil chemical properties

**Figure 4:**
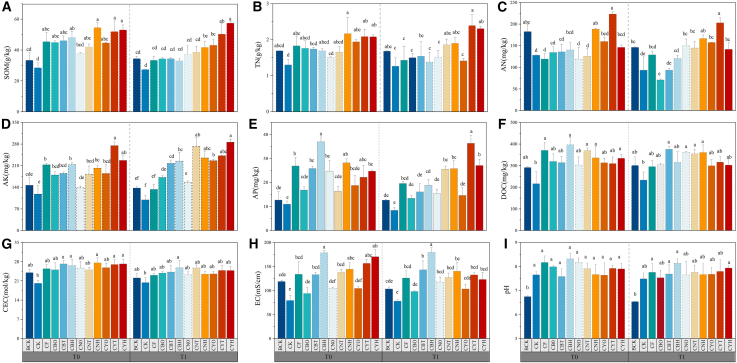
Effects of soil layer replacement and fertilization on soil chemical nutrients (A–I) Effects of soil layer replacement combined with organic fertilizer application on soil organic matter (A), total nitrogen (B), alkaline dissolved nitrogen (C), available potassium (D), available phosphorus (E), dissolved organic carbon (F), cation exchange capacity (G), electrical conductivity (H), and soil acidity (I). Data are presented as mean ± standard deviation (SD). Different lowercase letters indicate significant differences among treatments at *p* < 0.05.

The application of organic fertilizers under the two soil replacement depths exerted a significant influence on soil fertility and led to an increase in the concentrations of nutrient indicators, including SOM, total nitrogen (TN), cation exchange capacity (CEC), and available phosphorus (AP) in the soil (Figure 4). Within the cow manure treatment groups, compared with the CNH and CNT treatments, the CNO treatment had no significant effect on most soil nutrients. This could potentially be attributed to the insufficient amount of cow manure applied. Cow manure can augment SOM and replenish the macro-elements, such as nitrogen, phosphorus, and potassium, essential for crop growth. Additionally, it supplies medium elements like calcium, magnesium, and sulfur, as well as trace elements including iron, manganese, Zn, Cu, and boron. These have a multifaceted positive impact on soil nutrients.^40^^,^^48^ Hence, it is of great significance to ensure that the quantity of fertilizers applied remains within a reasonable range to avoid a reduction in crop yield resulting from discrepancies in the fertilizer application rate.

Under the two soil layer replacement conditions, compared with other organic fertilizers treatments, the CYH and CNH treatments exhibited a better overall effect on soil nutrients, with a particularly notable effect on SOM and AP. Under T0 condition, compared with the CK, the SOM concentrations in the CYH and CNH treatments increased by 47.75% and 46.32%, respectively (Figure 4A), while the AP content increased by 157% and 125%, respectively (*p* < 0.05) (Figure 4E). Under the T1 condition, compared with CK, the SOM concentrations in the CYH and CNH treatments increased by 34.44% and 52.23%, respectively (Figure 4A). Meanwhile, the AP concentrations increased by 148% and 221%, respectively (Figure 4E). Significant changes in soil pH occur after soil layer replacement (Table S1). Although the capability of biochar treatments (CBO, CBT, and CBH) to enhance soil nutrients is less pronounced than that of the liquor brewing sludge-derived organic fertilizer treatments (CYO, CYT, and CYH) and cow manure treatments (CNO, CNT, and CNH), it can effectively elevate the pH of soil. The soil under the biochar treatments was slightly alkaline (Figure 4I), and compared with the CK treatment, the CBH treatment showed the largest increase (T0: 0.67 units; T1: 0.40 units). The soil in the southwest karst region is acidic, influenced by factors such as topography, climate conditions, soil-forming parent materials, and mining activities. Nevertheless, the minerals in the biochar raw materials can form alkaline substances, such as potassium, calcium, and magnesium oxides and hydroxides. These alkaline substances react with hydrogen ions in the soil to neutralize them, thereby reducing the concentration of these ions in the soil.^49^ As a result, the soil pH is increased, and the acidic substances in the soil are fundamentally consumed, which can significantly improve the acidic soil conditions.^49^^,^^50^

Notably, the long-term maintenance and improvement of soil fertility after soil layer replacement are highly dependent on the continuous input of organic fertilizer. The organic fertilizer applied in this study can steadily replenish SOM and nutrients, provide effective substrates for microbial activities, promote the formation of soil aggregates, and enhance the CEC, thereby achieving the long-term supply of soil fertility. However, the organic matter incorporated into soil tends to be gradually mineralized and decomposed over time, and its long-term effects on soil organic carbon sequestration, nutrient cycling, and fertility sustainability still need to be further verified through long-term field experiments.

### Soil microbial properties

**Figure 5:**
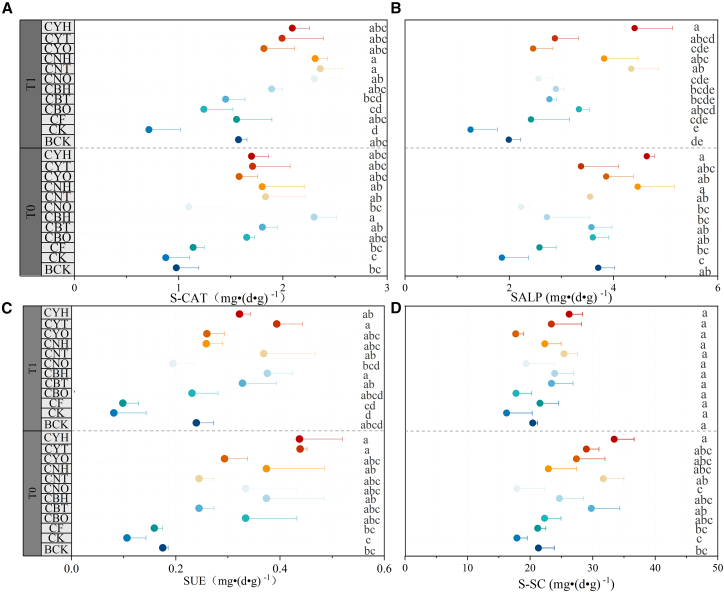
Effects of soil layer replacement combined with organic fertilizer application on microbial properties (A–D) Effects of soil layer replacement combined with organic fertilizer application on soil catalase (A), soil alkaline phosphatase (B), soil urease (C), and soil sucrase (D). Data are presented as mean ± standard deviation (SD). Different lowercase letters indicate significant differences among treatments at *p* < 0.05.

In terms of soil microbial activity, soil enzyme activity serves as a crucial indicator for assessing soil quality.^51^ The combined application of organic fertilizers following soil replacement induced remarkable changes in the activities of soil sucrase (S-SC), soil catalase (S-CAT), soil urease (SUE), and soil alkaline phosphatase (SALP) (Figure 5). Under T0 condition, compared with those in the CK, the soil enzyme activity indicators, S-SC, S-CAT, SUE, and SALP, under the CYH treatment increased significantly. Specifically, the levels of these indicators increased by 86%, 155%, 150%, and 25%, respectively. Under T1 condition, compared with the CK treatment, the activities of soil enzymes (S-CAT, SALP, SUE, and S-SC) in the CNT, CYH, and CYT treatments all showed an increase. The ranks of the treatment groups in terms of their effect on improving soil enzyme activity are as follows: liquor-brewing sludge organic fertilizers > cow manure > biochar > chemical fertilizers > blank control. The CYH treatment among the liquor-brewing sludge-based treatment groups proved to be the most effective fertilization method for enhancing soil microbial activity. This effectiveness mainly originated from two key aspects. Firstly, the soil treated with liquor brewing sludge-derived organic fertilizers presented an optimal pore structure. Such a pore configuration was highly favorable for the migration and spread of microorganisms within the soil matrix, thereby significantly boosting the activity of these microorganisms.^52^^,^^53^ Secondly, the liquor brewing sludge-derived organic fertilizers are abundant in a wide variety of organic components, such as carbohydrates, proteins, and fats. These components could be easily decomposed and assimilated by microorganisms, thus being transformed into their biomass and energy sources. This process not only promoted the proliferation and metabolic activities of microorganisms but also brought about changes in the structure of the microbial community.^27^^,^^54^ Moreover, liquor brewing sludge-derived organic fertilizers contain potassium-solubilizing bacteria and microorganisms capable of secreting growth hormones. These microorganisms could act as substrates or activators for soil enzymes, effectively increasing the catalytic efficiency of these enzymes.^43^^,^^55^

### Soil quality evaluation

**Figure 6:**
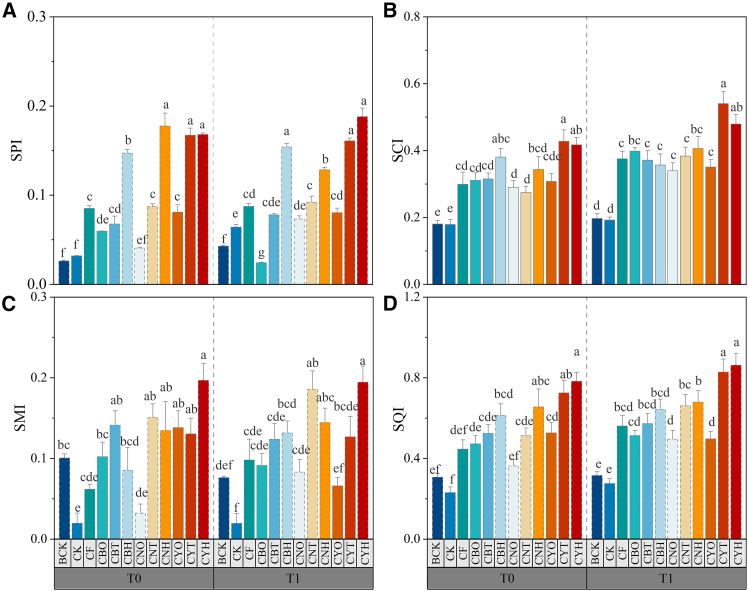
Effects of soil layer replacement combined with organic fertilizer application on soil quality indexes in different soil replacement depths (A–D) Effects of soil layer replacement combined with organic fertilizer application on soil physical property index (A), soil chemical property index (B), soil microbial property index (C), and soil quality index (D). Data are presented as mean ± standard deviation (SD). Different lowercase letters indicate significant differences among treatments at *p* < 0.05.

The weighted values derived from the principal-component analysis (PCA) and soil indicator scores were employed to calculate the SPI, SCI, SMI, and SQI (Figure 6). The discrepancies among the treatments under the two soil replacement modes generally attained a significant level. Under T0 condition, across different fertilization treatments, the SQI values ranged from 0.23 to 0.78. When contrasted with the CK and CF treatments, the SPI, SCI, SMI, and SQI values of the CYH, CNH, CYT, and CBH treatments displayed higher significance (*p* < 0.05). The CYH treatment demonstrated the highest comprehensive values across the four quality assessment indicators. SPI, SCI, SMI, and SQI values for the CYH treatment were 0.16, 0.41, 0.19, and 0.78, respectively. Under T1 condition, the SQI values ranged between 0.27 and 0.86 (Figure 6D). The SPI, SCI, SMI, and SQI values under the CYH treatment were 0.18, 0.47, 0.19, and 0.86, respectively. The soil properties in the CYH treatment group showed significant superiority over those in the other groups, demonstrating favorable physical, chemical, and microbial characteristics.

### Maize yield and SPI, SCI, SMI, and SQI relationships

**Figure 7:**
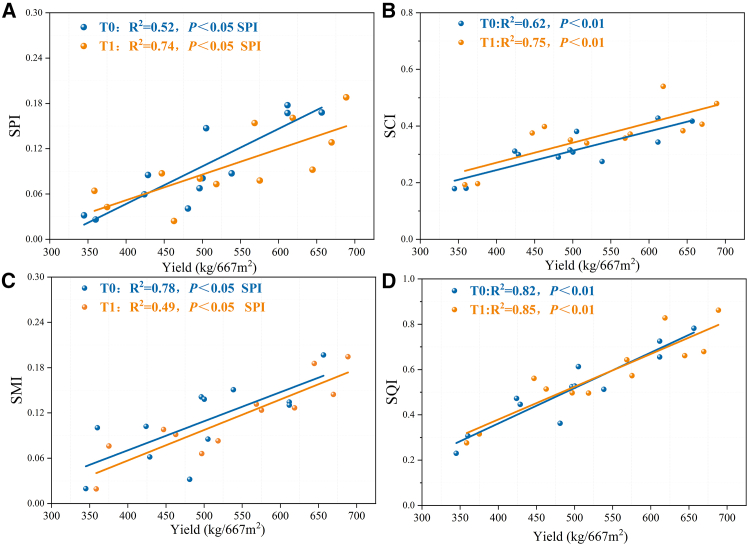
Correlation fitting between maize yield and soil quality index under different soil layer replacement depths (A–D) Correlation fitting between maize yield and soil physical property index (A), soil chemical property index (B), soil microbial property index (C), and soil quality index (D) under different soil layer replacement depths. Pearson’s correlation analysis was used, and the significance level was set at *p* < 0.05 and *p* < 0.01.

**Figure 8:**
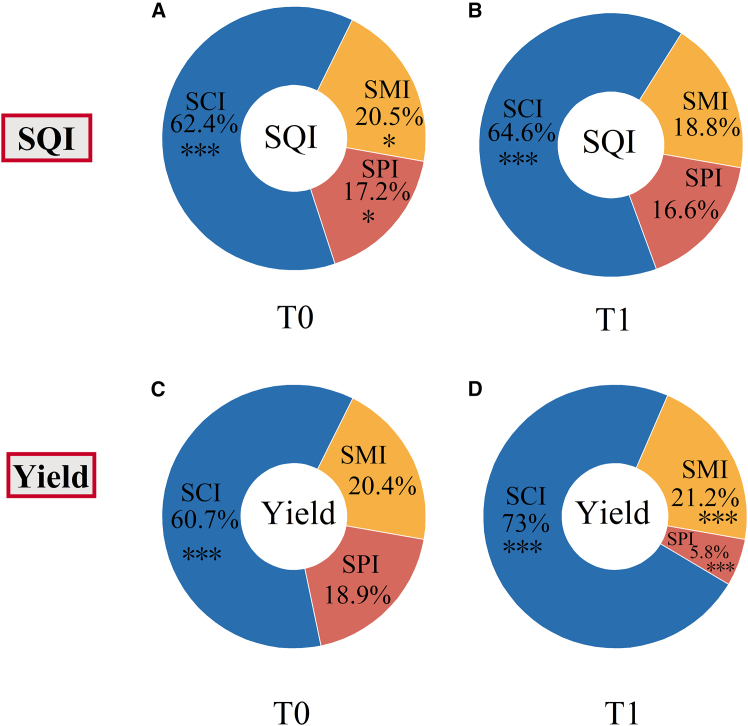
Contributions of SPI, SCI, and SMI to SQI and maize yield under different soil replacement depths (A) Contributions of SPI, SCI, and SMI to SQI under T0 soil replacement depths. (B) Contributions of SPI, SCI, and SMI to SQI under T1 soil replacement depths. (C) Contributions of SPI, SCI, and SMI to yield under T0 soil replacement depths. (D) Contributions of SPI, SCI, and SMI to yield under T1 soil replacement depths. Statistical significance was determined using one-way ANOVA, followed by Duncan’s multiple range test. ∗, ∗∗, and ∗∗∗ indicate significant differences at *p* < 0.05, *p* < 0.01, and *p* < 0.001, respectively.

Soil layer replacement combined with organic fertilizer application exerted significant effects on soil quality and maize yield (Figure 7). The overall magnitude of yield increase among the treatments followed the order as follows: conventional chemical fertilizer < cow manure < biochar < liquor brewing sludge-derived organic fertilizers. Compared with CK, the application of liquor brewing sludge-derived organic fertilizers increased maize yield by more than 38.0%. Correlation analysis revealed that SPI, SCI, SMI, and SQI were all significantly correlated with maize yield. Under T0 treatment, maize yield showed extremely significant positive correlations with SQI and SCI (R^2^ = 0.82, *p* < 0.01; R^2^ = 0.62, *p* < 0.01) (Figure 7D). SCI contributed the most to SQI, reaching 62.4%, followed by SMI, while SPI exhibited the lowest contribution (Figure 8A). Under T1 treatment, maize yield was also significantly and positively correlated with the SQI and SCI (R^2^ = 0.85, *p* < 0.01) (Figure 8D). Under both soil replacement depths, SCI displayed the highest coefficient of determination for maize yield, indicating that soil chemical properties were a key factor limiting maize production. Due to the inherent characteristics of shallow soil layers and poor nutrient content in karst areas, soil layer replacement further exacerbates the problem of insufficient available nutrients, making soil chemical nutrients (especially organic matter, nitrogen, phosphorus, etc.) the most direct factor restricting crop growth. Under the specific environmental background of this region, although soil physical and microbial properties have a positive effect on soil quality, their contribution to corn yield is relatively limited. Therefore, soil chemical properties have become the core limiting factor affecting yield.

### Effects of soil properties on SQI and maize yield

**Figure 9:**
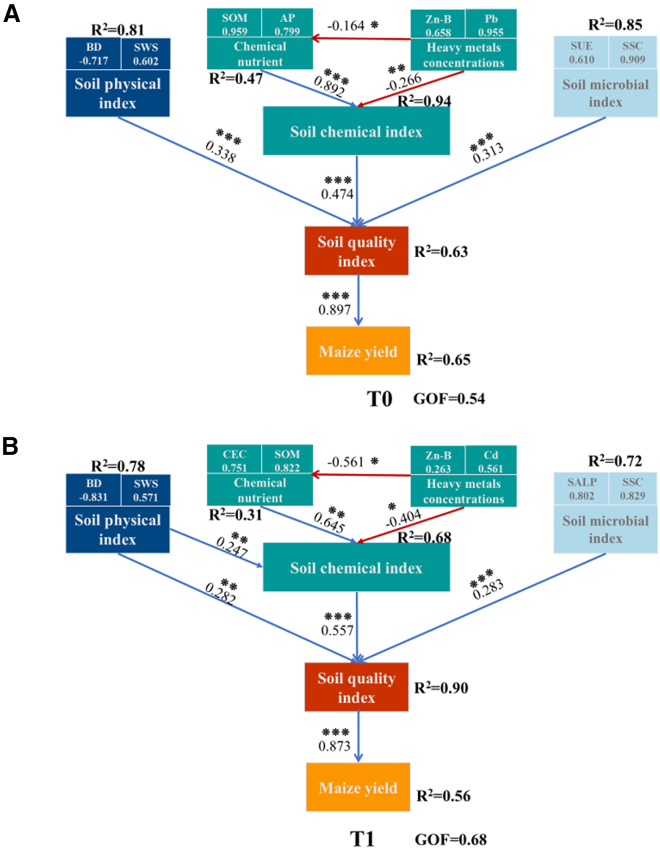
PLS-PM revealing the effects of different soil replacement depths on soil quality index and maize yield (A and B) Path analysis under T0 (A) and T1 (B) treatments. Values above lines represent the standardized path coefficients, and asterisks denote the significance levels. Blue lines indicate positive effects, and red lines indicate negative effects. R^2^ represents the coefficient of determination, and GOF denotes the goodness-of-fit of the model. PLS-PM analysis was performed using Smart PLS 4.0. ∗, ∗∗, and ∗∗∗ indicate significant differences at *p* < 0.05, *p* < 0.01, and *p* < 0.001, respectively.

Partial least-squares path modeling (PLS-PM) analysis was performed to explore the interconnections among the two soil layer replacement modes, fertilizers application, soil quality indices (SPI, SCI, SMI, and SQI), and maize yield. In both soil layer replacement modes, SPI, SCI, and SMI exerted direct or indirect effects on the SQI and maize yield (Figure 9). Under T1 condition, the SPI, SCI, and SMI had significant positive effects on SQI (standardized path coefficients were 0.282, 0.557, and 0.283, respectively; *p* < 0.01), and SQI had a positive effect on maize yield as well (standardized path coefficient = 0.873; *p* < 0.001) (Figure 9B). Notably, soil heavy metals had a direct negative effect (*p* < 0.001) on soil chemical nutrient indices under the two soil replacement modes. The probable reason lies in the fact that heavy metals are capable of engaging in chemical reactions with soil nutrients, thereby forming insoluble complexes.^56^ For example, Cd and Pb can bind to phosphate ions, resulting in the formation of precipitates such as cadmium phosphate and lead phosphate. This process diminishes the availability of the nutrient element phosphorus in the soil,^57^ impacts the absorption and transport of nutrients, and ultimately gives rise to a nutrient imbalance within plants.

In conclusion, different replacement depths directly influence soil quality and ultimately maize yield by directly affecting physical, chemical, and microbial properties.^4^ Under the T1 replacement condition, the model’s explanatory power for SQI (R^2^ = 0.90) was significantly higher than that under T0 (R^2^ = 0.63). SPI, SCI, and SMI under the T0 and T1 treatments accounted for only 54% and 68% of the variation in SQI and maize yield, respectively. This was because the variation in maize yield is influenced by the variety characteristics, planting management practices, climatic conditions, pests, and diseases,^58^ rather than being solely determined by the linear relationships among SPI, SCI, SMI, SQI, and maize yield. Long-term monitoring should be carried out to assess the interrelationships among field management, corn growth indicators, soil quality, and maize yield.

### Conclusion

This field-based experimental study demonstrated that the application of liquor brewing sludge-derived organic fertilizers significantly promoted the decomposition of organic matter and the nutrient cycling processes within the soil. It exerted a profound influence on the physical, chemical, and microbial properties of the soil, thereby serving as an effective field fertilization approach for enhancing soil quality and boosting maize yield. The application of organic fertilizers exerted differential effects on soil properties across different soil layer replacement depths. Compared with replacement depths of 0–20 and 20–40 cm, treatments with 0–30 cm and 30–60 cm soil layer replacement exhibited lower Cd, Pb, and Zn concentrations that were below the threshold values specified in the Risk Control Standard for Soil Contamination of Agricultural Land (GB 15618-2018). Moreover, these treatments achieved a more pronounced reduction in both total and bioavailable concentrations of heavy metals in surface soils, indicating that this strategy represents an effective measure for alleviating heavy metal pollution in the cultivated soil layer. The evaluation system composed of SPI, SCI, SMI, and SQI allows for a systematic and comprehensive assessment of soil physical, chemical, microbial, and soil environmental quality and enables accurate identification of the key driving factors for crop yield. The SCI values under the soil layer replacement of 0–30 and 30–60 cm were generally higher than those under the replacement of 0–20 and 20–40 cm soil layers. SCI makes the greatest contribution to SQI, followed by SMI, with SPI contributing the least. SQI exhibited the most direct influence on maize yield, with SCI being the primary contributing factor. Both heavy metal total concentrations and bioavailable concentrations have a negative effect on the SCI. Furthermore, long-term field experiments are crucial for comprehensively evaluating the integrated effects of soil replacement combined with organic fertilizer application on heavy metal reduction, soil quality improvement, and maize yield enhancement. The research results can provide a scientific basis for policies related to resource utilization of solid waste in the liquor brewing industry and the safe utilization of heavy metal-contaminated cultivated land in karst areas.

### Limitations of the study

The field experiment was conducted only for two years without long-term located monitoring; so, the long-term sustainability of soil quality improvement, the stability of heavy metal passivation, and the continuous performance of maize yield under the combined practice of soil layer replacement and organic fertilizer application remain unclear. This study adopted fixed tillage and replacement depths as well as concentration gradients of organic fertilizer, without considering the spatial heterogeneity of heavy metal pollution, characteristics of combined multi-metal pollution in actual farmland, and the influences of different climatic conditions and agronomic management measures on remediation effects, which limits the regional popularization and applicability of the research findings. This research was analyzed merely from the perspectives of soil physicochemical properties, enzyme activities, heavy metal available fractions, and crop yield, while the microscopic mechanism underlying heavy metal migration and transformation as well as the response rules of soil microbial community were not further revealed. Meanwhile, the long-term environmental risk of heavy metal reactivation after remediation remains to be further explored.

## Resource availability

### Lead contact

Requests for further information and resources should be directed to and will be fulfilled by the lead contact, Xuexian Li (xxli5@gzu.edu.cn).

### Materials availability

This study did not generate new unique reagents.

### Data and code availability

• The data supporting the findings of this study are available within the article and supplemental information.
• This paper does not report any original code.
• Any additional information required is available upon reasonable request to the lead contact.

## Acknowledgments

This study was financially supported by the High-Level Talent Training Program in Guizhou Province (GCC [2023] 045).

## Author contributions

Conceptualization, X.L.; data curation, H.W., H.T., and X.L.; investigation, H.W. and H.T.; visualization, H.W.; writing – original draft, H.W.; writing – review & editing, P. Wang, X.L., and P. Wu; resources, H.T.; supervision, X.L. and P. Wu; project administration, X.L. and P. Wu; funding acquisition, P. Wu.

## Declaration of interests

The authors declare no competing interests.

## STAR★Methods

### Key resources table

**Table:** 

REAGENT or RESOURCE	SOURCE	IDENTIFIER
**Chemicals, peptides, and recombinant proteins**		

NaOH	Chronchem	1310-73-2
K_2_CrO_7_	Chronchem	7778-50-9
NaHCO_3_	Chronchem	144-55-8
HCl	Chronchem	7647-01-0
HNO_3_	Chronchem	7697-37-2
HF	Chronchem	7664-39-3
HClO_4_	Chronchem	7601-90-3
H_2_SO_4_	Chronchem	7664-93-9

**Software and algorithms**		

Smart PLS 4.0	Smart PLS GmbH	https://www.smartpls.com
SPSS 26.0	IBM Corporation	https://www.ibm.com/products/spss
OriginPro 2023	OriginLab Corporation	https://www.originlab.com

**Other**		

Cow manure	Weining County, Bijie City, Guizhou Province	N/A
Biochar	Yunnan Yuntianhua Co., Ltd	N/A
Liquor brewing sludge-derived organic fertilizer	Zunyi Nanhuan Environmental Protection Technology Co., Ltd	N/A

### Method details

#### Overview of the study area

The experimental site was conducted in a typical heavy metals pollution area caused by high geological background and historical lead-zinc smelting activities, located in Weining County (26°36′32″N, 104°42′22″E), southwestern China. The study area is a typical karst region with extensive distribution of carbonate rocks. The regional soil generally shows enrichment of heavy metals such as Cd, Pb, Zn, Ni, and Cr.^59^ Among them, Cd and Pb have a high degree of enrichment, and their background values have exceeded the limits specified in the Soil Environmental Quality Standard (GB 15618-2018).^5^^,^^6^ Historical Pb-Zn mining and smelting activities have intensified the migration, release, and accumulation of heavy metals into the surficial environment.^60^ The superimposition of natural geological background and mining-smelting sources has resulted in significant and widespread accumulation of heavy metals such as Cd and Pb in the surface soil of regional cultivated land.^10^

The study area belongs to the plateau mountainous temperate climate, with an average annual temperature of 12.5°C and low precipitation, with an annual rainfall of 790–910 mm. Constrained by the karst landform, the regional ecological environment is fragile, cultivated land resources are scarce and fragmented, soil layers are shallow, water retention capacity is poor, soil maturity is low, and nutrients are deficient, mainly in dryland. The zonal soil is dominated by yellow-brown soil, accounting for about 65% of the regional area. Traditional agricultural planting is mainly composed of maize, potato, and soybean, among which maize has become the most important economic crop due to its drought resistance and tolerance, with a perennial planting area of about 1334 hm^2^. Additionally, the fragmentation of cultivated land, heavy metals pollution, and low soil nutrient levels in the study area have become key factors restricting the efficient and sustainable development of regional agriculture, forming a typical agricultural development dilemma of “high environmental risk-low production efficiency” in karst mountainous areas.

#### Experimental design

We employed mechanical equipment to conduct reciprocal soil layer replacement for two plowing modes: one involving the 0–20, 20–40 cm soil layers, and the other involving the 0–30, 30–60 cm soil layers. Soil layer replacement refers to the process of exchanging less heavy metals-contaminated deep soil with more heavily contaminated shallow soil.^17^ After plowing, topsoil samples (0–20 cm) were collected, and their basic physicochemical properties are presented in Table S2. This two-year field experiment was designed as a randomized block design with 3 replicates, including two plowing modes and 12 treatment groups, resulting in a total of 72 experimental plots. The 12 treatments comprised no replacement + no fertilization (BCK), blank control (CK), conventional chemical fertilization (CF), and three concentration gradients (low, medium, high) of three organic fertilizers: biochar (CBO, CBT, CBH), cow manure (CNO, CNT, CNH), and liquor brewing sludge-derived organic fertilizer (CYO, CYT, CYH). Detailed fertilization schemes for all treatments are provided in Table S3. The preparation process of the liquor brewing sludge-derived organic fertilizer is as follows: liquor sludge (moisture concentration 60%–80%), pit mud (moisture concentration 50%), auxiliary materials, and functional microbial inoculants are thoroughly mixed, followed by controlled aerobic fermentation for approximately 10 days with regular turning and moisture adjustment to ensure sufficient decomposition and stabilization; the final product is characterized by high organic matter concentration, a pH of 7–9, a porosity of 30–40%, and a carbon-to-nitrogen ratio (C/N) of 20–30. Then, the mixture undergoes continuous tank fermentation to fully decompose the organic materials. The types of fertilizers and the amounts of fertilization are shown in Table S4. The maize cultivar tested in this experiment was “Dunyu 810”, a common variety adopted by local farmers. All fertilizers were applied as base fertilizers in one application before sowing. All other field management practices adhered to local standard cultivation protocols.

#### Sample collection and testing methods

During the maize grain maturity stage, six representative maize plants were randomly selected from each plot for threshing. The grains were then dried at 60 °C to a constant weight, following which their moisture concentration was measured. Finally, the grain weight was converted to maize grain yield on a dry-weight basis. The surface soil (0–20 cm) was collected via the five-point sampling method, and debris, including gravel and roots, was removed. The soil was naturally air-dried in a cool place, passed through 2 mm and 0.149 mm sieves, and then sealed for storage. The soil pH and electric conductivity (EC) were determined using the potentiometric method (NY/T 1377–2007), the bulk density (BD) was measured by the ring knife method, and the water concentration was determined by the gravimetric method.^61^ The traditional method used to determine soil organic carbon (SOC), total nitrogen (TN), available nitrogen (AN), available phosphorus (AP), and available potassium (AK).^17^ Dissolved organic carbon (DOC) was analyzed and determined by a TOC/TN analyzer.^62^ The cation exchange capacity (CEC) of soil was measured using the ammonium acetate method.^63^ Determination of the activities of four enzymes, soil sucrase, soil catalase, soil urease, and soil alkaline phosphatase, using a 96-μL fluorometric assay.^64^ The activities of the aforementioned soil enzymes were all measured using reagents (Beijing Solaibao Technology Co., Ltd.).

To determine the total amount of soil heavy metals, HNO_3_ and HF (3:1) were added to a dry and sealed polytetrafluoroethylene digestion tank and kept at a temperature of 180 °C for 48 h. After cooling, the digestion tank was taken out and placed on an electric hot plate at 120 °C to remove the acid until the solution became viscous. Then, it was diluted and made up to 50 mL with 2% HNO_3_, left to stand for 1 h, and the concentrations of Cd, Pb, and Zn were determined using an inductively coupled plasma mass spectrometer (ICP-MS; Thermo Fisher X Series 2).^59^ During the analysis, quality control was implemented using reagent blanks, 20% parallel samples, and the national standard soil sample GSS-5. The spiked recovery rates of heavy metals ranged from 110% to 120%, which met the established quality control standards.

#### Soil quality index (SQI) evaluation

##### Index scoring of the minimum dataset (MDS)

The Soil Quality Index (SQI) is an indicator system for comprehensively evaluating the soil quality status.^2^ Based on the soil management assessment framework, a Total DataSet (TDS) was constructed, and principal components (PCA) with eigenvalues >1 were incorporated into the Minimum DataSet (MDS), retaining only absolute numerical variables within the top 10% of maximum loading coefficients (Table S5). When a principal component contained multiple variables, only indicator pairs with correlation coefficients <0.6 were retained. Principal component analysis (PCA) and correlation analysis are used to analyze and standardize the soil characteristic indicators.^33^ The measured indicator values are transformed into dimensionless values between 0 and 1.^2^

For soil nutrient indicators, the “more is better” function Equation 1 is used to calculate the scores. For indicators that have an adverse impact on soil quality, such as the concentration of heavy metals, the “less is better” function Equation 2 is used to calculate the scores. For nonlinear indicators like soil pH and soil bulk density (BD), the “optimum” function Equation 3 is used to calculate the scores. The function thresholds x_1_ and x_2_ for pH are 6.0 and 8.0, respectively, and the function thresholds x_1_ and x_2_ for BD are 0.9 and 1.3, respectively.^65^

(Equation 1) $$S=1+0.9\times \frac{\left(x-x_{\min}\right)}{\left(x_{\max}-x_{\min}\right)},x_{\min}\leq x\leq x_{\max}$$

(Equation 2) $$S=1-0.9\times \frac{\left(x-x_{\min}\right)}{\left(x_{\max}-x_{\min}\right)},x_{\min}\leq x\leq x_{\max}$$

(Equation 3) $$S=0.1+0.9\times \frac{\left(x-x_{1}\right)}{\left(x_{2}-x_{1}\right)},x_{1}\leq x\leq x_{2}$$

Where S represents the index score, x represents the measured value of the index.

##### SQI calculation

Upon completion of weighting and scoring the soil MDS indicators, SQI, SPI, SCI, and SMI were computed. The calculation of SPI, SCI, and SMI to SQI system algorithm enables a more nuanced representation of how distinct indicators influence soil quality and maize yield.^2^

(Equation 4) $$\text{SQI}\;\left(\text{SPI},\text{SCI},\text{SMI}\right)=\sum_{i=1}^{n}\;\text{Wi}\times \text{Si}$$

Where Wi represents the index weight value (the ratio of the common factor variance of the corresponding index to the sum of the common factor variances of all indices), and Si represents the standardized score of the index.

### Quantification and statistical analysis

Data processing, statistical analysis, and plotting were performed using Excel 2010 and Origin 2021. Analysis of variance (ANOVA) was conducted with SPSS 19.0 to examine the effects of soil layer replacement and fertilization on soil physicochemical properties, soil quality indices (SQI, SPI, SCI, SMI), and maize yield. Significance differences were marked with asterisks, with significance levels defined as ∗*p < 0.05*, ∗∗*p < 0.01*, ∗∗∗*p < 0.001*. Duncan’s multiple range test was used for mean comparisons. Linear regression was applied to identify key factors influencing SQI and maize yield under different soil replacement depths. The partial least squares path model (PLS-PM) was used to analyze the relationships among SQI, SPI, SCI, SMI, and maize yield, and the model was implemented using Smart PLS 4.0 software.
