# Supplementary material for: Effects of soil layer replacement combined with organic fertilizer application on soil quality and maize yield in heavy metal-contaminated farmland
Source: iScience. 2026 Jun 12;29(7):116381. doi: 10.1016/j.isci.2026.116381 (PMC13277532; doi:10.1016/j.isci.2026.116381)
Supplement: Document S1. Figure S1 and Tables S1–S5 [file mmc1.pdf]

iScience, Volume *29*

Supplemental information

Effects of soil layer replacement combined with organic fertilizer application on soil quality and maize yield in heavy metal-contaminated farmland

Huanhuan Wang, Han Tu, Ping Wang, Xuexian Li, and Pan Wu

Supplementary Materials

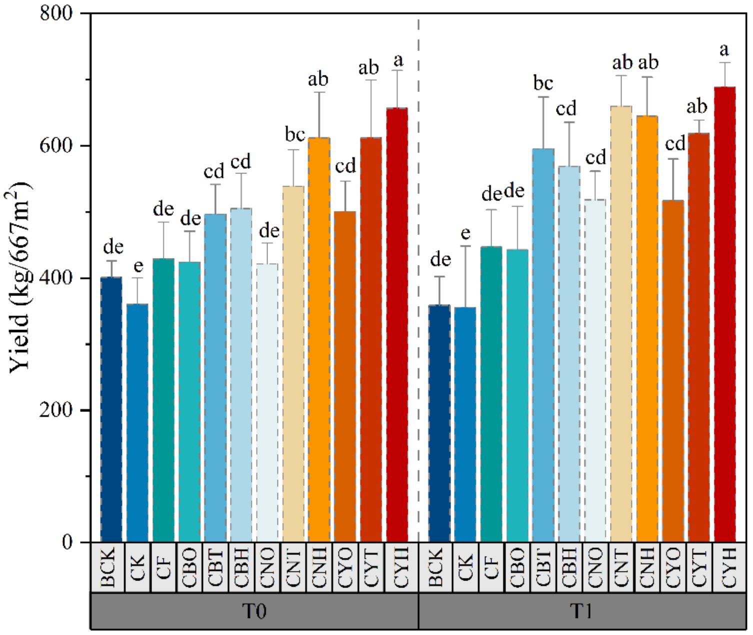

Figure S1. Effects of organic fertilizer application on maize yield under two soil layer replacement depths. T0: 0–20 cm and 20–40 cm replacement; T1: 0–30 cm and 30–60 cm replacement. BCK: no replacement and no fertilization; CK: blank control; CF: conventional chemical fertilization. Three application rates were set for each organic fertilizer: biochar (CBO, CBT, CBH), cow manure (CNO, CNT, CNH), and liquor brewing sludge-derived organic fertilizer (CYO, CYT, CYH). Data are presented as mean ± standard deviation (SD). Different lowercase letters indicate significant differences among treatments at $P < 0.05$.

Table S1. Effect of soil profile depth on soil pH

Depth of profile (cm)	pH
0-20	6.70
20-40	7.76
40-60	7.49

Table S2. Soil basic physicochemical properties

Soil replacement modes		pH	TN	SOM	AN	AK	AP	Cd	Pb
			(g/kg)		(mg/kg)				
0-20,20-40cm replacement	Before replacement	6.75	1.69	33.41	182.56	146.40	12.7	5.47	251
	After replacement	7.65	1.30	28.50	128.10	117.97	10.95	3.84	102
0-30,30-60cm replacement	Before replacement	6.53	1.68	35.57	146.3	137.50	12.63	5.53	306
	After replacement	7.53	1.26	27.4	108.61	99.36	8.42	3.03	108

Table S3. Different fertilization treatment designs in this study

Soil replacement modes	Treatments	Chemical nutrients (kg ha^{-1})			Organic nutrients (kg ha^{-1})		
		N	P_2O_5	K_2O	N	P_2O_5	K_2O
Not replaced	BCK	0	0	0	0	0	0
	CK	0	0	0	0	0	0
	CF	150	40	100	0	0	0
	CBO	0	0	0	125	46	467
	CBT	0	0	0	150	55	560
0-20cm, 20-40cm replacement	CBH	0	0	0	175	64	653
	CNO	0	0	0	125	60	93
	CNT	0	0	0	150	72	112
	CNH	0	0	0	175	84	131
	CYO	0	0	0	125	212	106
	CYT	0	0	0	150	255	128
	CYH	0	0	0	175	298	149
Not replaced	BCK	0	0	0	0	0	0
	CK	0	0	0	0	0	0
	CF	150	40	100	0	0	0
	CBO	0	0	0	125	46	467
	CBT	0	0	0	150	55	560
	CBH	0	0	0	175	64	653
0-30cm, 30-60cm replacement	CNO	0	0	0	125	60	93
	CNT	0	0	0	150	72	112
	CNH	0	0	0	175	84	131
	CYO	0	0	0	125	212	106
	CYT	0	0	0	150	255	128
	CYH	0	0	0	175	298	149

BCK, no replacement + no fertilization; CK, blank control; CF, conventional chemical fertilization; CBO, CBT, CBH, three concentration levels of biochar; CNO, CNT, CNH, three concentration levels of cow manure; CYO, CYT, CYH, three concentration levels of liquor brewing sludge-derived organic fertilizers.

Table S4. Fertilizers types

Fertilizers types	pH	TN	SOM	AN	AK	AP	Cd	Pb	Reference standard
		(g/kg)		(mg/kg)					
Chemical fertilizers	5.43	94.3	25.3	2157	186	24734	-	-	-
Biochar	8.34	18.8	35.5	146	137	12	2	306	GB/T 28731-2012
Cow manure	7.62	17.3	358	1050	51	858	4	17	-
Liquor brewing sludge-derived organic fertilizers	9.28	12.5	327	482	63	460	1	34	NY/T 525-2021

"-" indicates that no specific reference standard is available for this parameter.

Table S5. PCA results for soil properties

	Soil indictors	0-20 cm, 20-40 cm replacement					0-30 cm, 30-60 cm replacement			
		PC1	PC2	PC3	PC4	PC5	PC1	PC2	PC3	PC4
Soil physical properties	Soil water storage	**0.795**	0.036	-0.204	0.489	-0.112	**0.861**	0.109	-0.336	0.107
	Bulk density	-0.743	-0.065	-0.113	-0.329	**0.449**	-0.594	-0.067	**-0.671**	0.189
	Total porosity	0.626	-0.555	-0.302	-0.23	-0.273	0.803	-0.131	-0.100	-0.411
Soil chemical properties	pH	0.729	-0.227	0.169	-0.194	-0.003	0.522	-0.669	-0.029	-0.323
	Soil organic matter	**0.879**	0.284	-0.507	0.194	-0.015	**0.954**	0.128	0.062	0.102
	Dissolved organic carbon	0.502	0.237	0.411	-0.335	**0.447**	0.656	0.000	0.581	0.186
	Total nitrogen	0.67	0.551	-0.435	0.18	0.023	0.776	0.471	-0.112	0.157
	Available nitrogen	0.528	0.586	-0.249	-0.249	-0.193	0.457	0.682	-0.416	-0.277
	Available potassium	**0.869**	0.243	0.07	0.163	0.17	0.828	0.134	-0.060	-0.076
	Available phosphorus	0.816	0.357	-0.221	0.065	-0.238	0.684	-0.150	**0.612**	-0.185
	Cation exchange capacity	0.905	0.075	0.302	-0.117	0.125	**0.887**	0.253	0.181	0.127
	Electrical conductivity	0.695	0.02	**0.649**	-0.089	-0.128	0.822	0.203	0.254	-0.128
	Cadmium	-0.636	**0.735**	0.044	0.01	-0.017	**-0.828**	0.601	0.118	-0.097
	Lead	-0.622	**0.721**	0.051	-0.095	0.04	-0.291	**0.892**	0.280	-0.065
	Zinc	-0.699	0.607	-0.031	-0.135	0.01	-0.253	**0.860**	0.405	0.028
	Cadmium (Bioavailable concentration)	-0.587	**0.659**	0.37	0.076	-0.231	-0.735	0.404	0.368	0.074
	Lead (Bioavailable concentration)	-0.332	-0.156	0.123	**0.758**	0.419	-0.187	0.688	0.044	-0.211
	Zinc (Bioavailable concentration)	-0.027	-0.15	**0.710**	0.371	-0.401	-0.018	**-0.775**	0.592	0.406
Soil microbial properties	Soil sucrase	**0.819**	0.317	0.237	0.234	0.142	0.693	0.086	-0.358	**0.427**
	Soil catalase	0.663	-0.031	0.098	-0.488	0.103	0.838	-0.075	0.172	0.089
	Soil urease	**0.796**	0.328	0.287	-0.061	-<br>0.063	0.836	0.164	-0.120	-0.201
	Soil alkaline phosphatase	0.783	0.087	-0.053	0.073	0.494	0.548	0.590	-0.253	**0.430**
